# Radiological Assessment of Charcot Neuro-Osteoarthropathy in Diabetic Foot: A Narrative Review

**DOI:** 10.3390/diagnostics15060767

**Published:** 2025-03-19

**Authors:** Antonio Mascio, Chiara Comisi, Virginia Cinelli, Dario Pitocco, Tommaso Greco, Giulio Maccauro, Carlo Perisano

**Affiliations:** 1 Department of Orthopedics and Geriatric Sciences, Catholic University of the Sacred Heart, 00136 Rome, Italy; antonio.mascio87@gmail.com (A.M.); virginiacinelli23@gmail.com (V.C.); greco.tommaso@outlook.it (T.G.); giulio.maccauro@policlinicogemelli.it (G.M.); carlo.perisano@policlinicogemelli.it (C.P.); 2 Department of Orthopedics and Rheumatological Sciences, Fondazione Policlinico Universitario A. Gemelli IRCCS, 00136 Rome, Italy; 3 Diabetes Care Unit, Endocrinology, University Hospital “A. Gemelli”, Catholic University of the Sacred Heart, 00136 Rome, Italy; dario.pitocco@policlinicogemelli.it; 4 Department of Life Sciences, Health, and Healthcare Professions, Link Campus University, 00165 Rome, Italy

**Keywords:** Charcot Foot, diabetes mellitus, osteoarthropathy, imaging, early diagnosis, foot and ankle disease

## Abstract

Charcot Neuro-Osteoarthropathy (CNO) is a debilitating complication predominantly affecting individuals with diabetes and peripheral neuropathy. Radiological assessment plays a central role in the diagnosis, staging, and management of CNO. While plain radiographs remain the cornerstone of initial imaging, advanced modalities such as Magnetic Resonance Imaging (MRI) and Computed Tomography (CT) have significantly enhanced diagnostic accuracy. Nuclear imaging, including bone scintigraphy, radiolabeled leukocyte scans, and FDG-PET/CT, offers additional diagnostic precision in complex cases, especially when differentiating CNO from infections or evaluating patients with metal implants. This review underscores the importance of a multimodal imaging approach suited to the clinical stage and specific diagnostic challenges of CNO. It highlights the critical need for standardized imaging protocols and integrated diagnostic algorithms that combine radiological, clinical, and laboratory findings. Advances in imaging biomarkers and novel techniques such as diffusion-weighted MRI hold promise for improving early detection and monitoring treatment efficacy. In conclusion, the effective management of CNO in diabetic foot patients requires a multidisciplinary approach that integrates advanced imaging technologies with clinical expertise. Timely and accurate diagnosis not only prevents debilitating complications but also facilitates the development of personalized therapeutic strategies, ultimately improving patient outcomes.

## 1. Introduction

Charcot Foot (CF), a distinct and severe manifestation within the broader spectrum of Charcot Neuro-Osteoarthropathy (CNO), is a chronic, progressive, and degenerative condition that primarily affects individuals with underlying peripheral neuropathy, most commonly due to diabetes mellitus [1]. This disorder is characterized by a gradual and often insidious loss of protective sensation in the foot and ankle, rendering patients unable to perceive minor injuries, mechanical stress, or repetitive microtrauma that would typically trigger a protective response [1].

The combination of sensory impairment and continuous, unrecognized trauma sets off a cascade of pathological events, ultimately leading to the progressive destruction of bones, joints, and periarticular structures within the foot and ankle complex [2]. As the disease advances, the affected bones undergo demineralization, fragmentation, and architectural distortion, resulting in joint instability, collapse, and severe deformities that significantly impair weight-bearing function and mobility. This pathological process, driven by a dysregulated inflammatory response and abnormal bone remodeling, further exacerbates structural deterioration and increases the risk of secondary complications such as ulceration, infection, and, in severe cases, limb amputation [2].

Charcot Foot represents one of the most serious and debilitating complications associated with diabetes and peripheral neuropathy, contributing to substantial morbidity and reduced quality of life for affected individuals [3]. The condition is often underdiagnosed in its early stages due to its nonspecific clinical presentation, which can mimic other inflammatory or infectious foot disorders. As a result, delayed recognition and intervention frequently allow for unchecked disease progression, leading to irreversible deformities that necessitate complex therapeutic approaches, ranging from conservative offloading strategies to reconstructive surgery [3].

Given its significant impact on patient outcomes, early identification and appropriate management of Charcot Foot are critical in mitigating long-term complications and preserving functional integrity. Advancements in diagnostic imaging, coupled with a multidisciplinary approach involving endocrinologists, podiatrists, orthopedic surgeons, and rehabilitation specialists, play a pivotal role in optimizing care for individuals with this devastating condition [2,3].

CNO is not exclusive to diabetes, as it is also linked to various neurological diseases, infections, and toxic syndromes, such as alcohol abuse [4]. The condition was first described in 1868 by Jean-Martin Charcot, who identified Charcot Foot as a complication in patients with tabes dorsalis (a myelopathy caused by syphilis). Later, in 1936, William Reilly Jordan was the first to connect CNO of the foot and ankle to diabetes [3]. The most commonly affected joints are the tarsal-metatarsal bones, metatarsophalangeal bones, and tibio-tarsal bones [5,6,7,8].

Historically, in the Eichenholtz classification [9] CNO is divided into three stages: bone dissolution (stage I), bone coalescence (stage II), and bone remodeling (stage III). Radiologically, the Eichenholtz classification is notably comprehensive and advantageous; however, its practical application is somewhat constrained. In clinical settings, the initial phase is regarded as active, whereas the coalescent and reconstructive phases are deemed quiescent or reparative [10,11]. Sella and Barrette [11] introduced stage 0, similar to stages I and II, but differs due to the absence of radiographic bone abnormalities, and the technetium 99 bone scan is markedly positive. Indium and gallium scans are normal [10,12].

Currently, two main classification systems are used for CF: the Modified Eichenholtz Classification [13] and the Brodsky Classification [14]. The Eichenholtz classification is based on clinical and radiographic findings and divides the disease into four progressive stages.

Stage 0, or the acute inflammatory phase, is marked by skin changes without radiographic evidence of bone abnormalities. Stage 1, or the fragmentation phase, shows radiographic evidence of bone destruction, dislocations, or subluxations. Stage 2, or the coalescence phase, involves the fusion of adjacent bone fragments and the formation of new periosteal bone. Finally, Stage 3, or the consolidation phase, features bone remodeling with possible significant residual deformities [15].

In contrast, the Brodsky [14] classification focuses on the anatomical distribution of the affected bone segments, identifying involvement of the tarsus and tarsometatarsal joint (type 1), the transverse tarsal joint and peritalar joints (type 2), and the tibio-talar joint (type 3a). Type 3b also involves the calcaneus with associated Achilles tendon insufficiency. This system subsequently was modified by Trepman et al. to include type 4—involves a combination of areas and type 5—occurs solely within forefoot [16].

In addition, it is necessary to report that the Sanders–Frykberg classification, according to Wukich, has excellent reliability [17]. Anatomical classification in relation to the foot joint involved and can be divided as follows: pattern I: phalanges, interphalangeals, and metatarsophalangeals; pattern II: tarsometatarsal joints; pattern III: cuneonavicular, talonavicular, and calcaneocuboid joints; pattern IV: talocrural joint; pattern V: posterior calcaneus involvement.

## 2. Clinical Features and Diagnosis

The acute phase of CNO is characterized by noticeable clinical signs, including swelling, warmth, erythema, and a significant rise in local skin temperature, which can range from 2 to 8 °C higher than the temperature of the contralateral, unaffected foot [18,19]. These thermal and inflammatory changes are indicative of heightened metabolic and inflammatory activity in the affected area. Notably, pain is absent in nearly 50% of cases, a finding attributed to the presence of diabetic peripheral neuropathy, which diminishes sensory perception in the extremities. Paradoxically, more than 75% of patients report some degree of discomfort or vague pain, even in otherwise insensate limbs, highlighting the complex interplay of neuropathy and local pathological changes [20,21]. Impaired neurological functions, such as the loss of deep tendon reflexes—most commonly observed with the Achilles reflex—are also frequently documented in this phase, serving as additional diagnostic clues [22]. Focal painful neuropathies are characterized by localized damage or dysfunction affecting either a single nerve root or an adjacent group of nerve roots, typically involving the feet and/or legs. In some cases, these neuropathies extend to one or both thighs, significantly impacting mobility and quality of life. They are frequently accompanied by profound muscle wasting and debilitating weakness, which can result in instability and an increased risk of falls. This condition is clinically referred to as proximal motor neuropathy or ‘diabetic amyotrophy.’ It arises primarily due to radiculo-plexopathy—an inflammatory or ischemic process involving the nerve roots and plexuses—or femoral neuropathy, which specifically affects the femoral nerve. Notwithstanding the severity of the initial presentation, these painful conditions typically follow a self-limiting course, with gradual recovery observed over a period from 6 to 18 months [23]. Peripheral arterial disease (PAD) resulting from ischemia is a major factor contributing to the complications associated with the diabetic foot [24]. Individuals with diabetes not only exhibit a higher prevalence of atherosclerosis, but the progression of the disease is also accelerated compared to non-diabetic individuals [25]. Despite the often-distinctive clinical manifestations of the acute phase, this stage frequently remains underdiagnosed or misdiagnosed, primarily due to overlapping symptoms with other conditions, such as cellulitis or deep vein thrombosis. The lack of early recognition significantly contributes to the rapid progression of CNO to chronic stages. These advanced stages are marked by pronounced deformities and structural changes, severely compromising foot function and biomechanics [26]. A hallmark deformity of advanced CNO is the so-called “rocker-bottom foot”, characterized by a prominent heel and a convexly rounded sole, which further underscores the severe alterations in foot architecture associated with the disease [27,28]. Diagnostic approaches involve the integration of clinical examination and advanced imaging modalities, such as radiography, computed tomography (CT), and magnetic resonance imaging (MRI). These techniques are particularly critical during the acute and fragmentation phases of the pathological process, where distinguishing between bone destruction caused by mechanical factors and that induced by infectious processes is essential. Radiography provides initial insights into structural changes, while CT offers detailed visualization of cortical bone alterations. MRI, with its superior soft tissue contrast, plays a pivotal role in detecting early signs of infection, edema, and marrow involvement, thereby allowing for a more comprehensive evaluation of the underlying condition. This combined approach enhances diagnostic accuracy, guiding timely and targeted therapeutic interventions [29,30,31].

## 3. X-Rays

**Figure 1:**
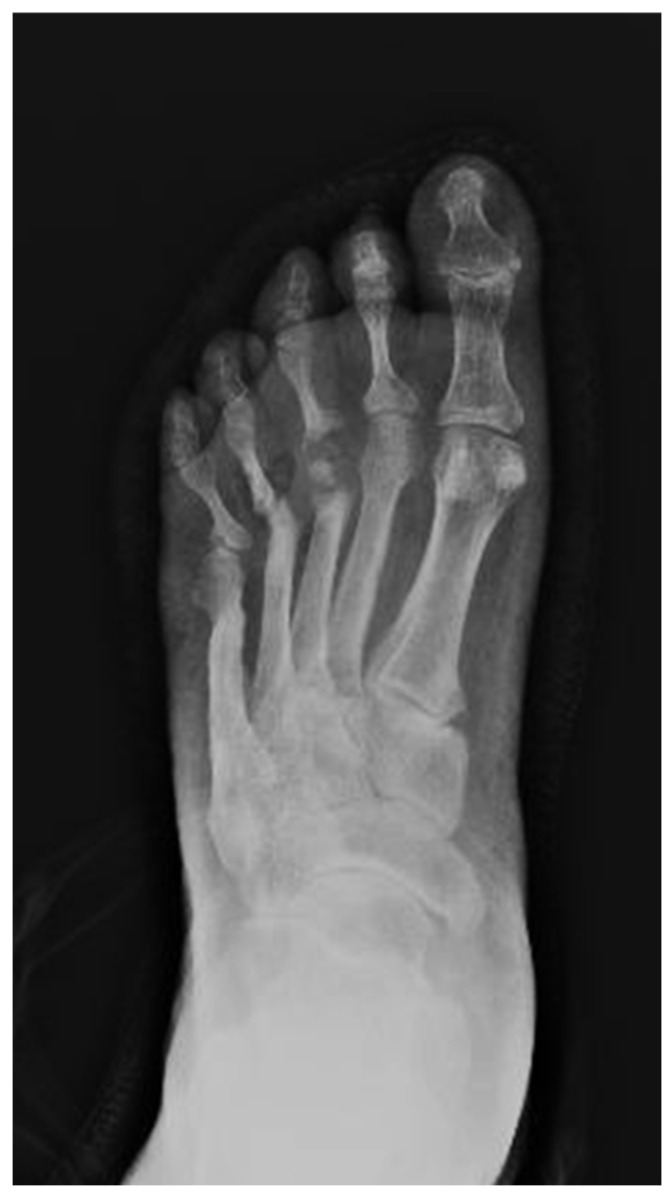
X-ray: Bony consolidation, fragmentation of the subchondral bone, fractures, dislocations, subluxations, osteopenia, and osteolysis.

Plain, lateral, dorsoplantar, and oblique weight-bearing radiographs represent a fundamental first-line diagnostic tool and are often indispensable in confirming the presence of CNO [32]. In the majority of cases, these imaging studies suffice to establish a definitive diagnosis, obviating the need for further advanced imaging modalities [33]. Radiographs provide characteristic findings in acute CN, including bony consolidation, fragmentation of the subchondral bone, fractures, dislocations, subluxations, osteopenia, and osteolysis, all of which are critical for accurate diagnosis and staging [34,35,36]. While there is some debate in the literature, weight-bearing radiographs performed without prior immobilization can be particularly useful in detecting subtle osseous changes, such as incipient fractures, early fragmentation, and joint subluxation (Figure 1). These findings are often more pronounced in the early stages of acute CNO compared to non-weight-bearing radiographs, which may fail to reveal these subtle pathologies [26,37].

Additionally, radiographic evaluation over time can provide insight into disease progression and response to treatment. Serial imaging allows clinicians to monitor the evolution of structural deformities and assess the effectiveness of therapeutic interventions, including surgical and nonsurgical approaches. Incorporating these radiographic findings into a comprehensive clinical assessment enhances diagnostic accuracy and facilitates the development of tailored treatment plans for patients with acute CNO [38,39].

The absence of abnormalities on initial radiographs does not necessarily rule out the presence of CNO. Consequently, further imaging studies may be essential to confirm the diagnosis [40,41]. Readily accessible and cost-effective, follow-up radiographs can serve as a crucial diagnostic tool, particularly in remote areas where advanced imaging technologies are unavailable. Typically, repeat X-rays are performed approximately two weeks after the initial examination, as radiographic indicators of acute CNO, such as bony changes and structural abnormalities, tend to become more pronounced over this time frame [42].

**Figure 2:**
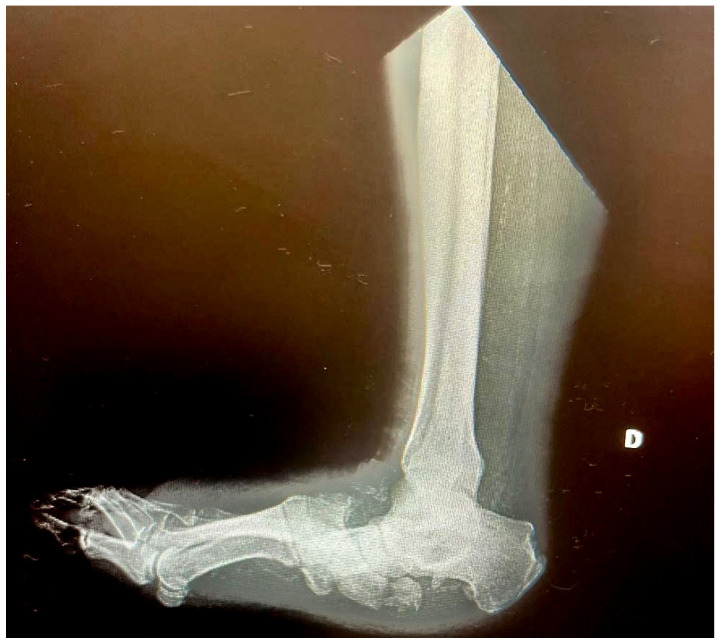
X-ray: Rocker-bottom deformity, dislocation of the tarsometatarsal joints from the second to the fifth rays, along with bony fragmentation and collapse of the midfoot.

Weight-bearing dorsoplantar and lateral foot radiographs often reveal several characteristic findings. These may include lateral dislocation of the tarsometatarsal joints from the second to the fifth rays, along with bony fragmentation and collapse of the midfoot, leading to the classic rocker-bottom deformity (Figure 2). Additionally, severe arthropathy of the tarsal bones with joint destruction is commonly observed, accompanied by diffuse bony sclerosis and remodeling [43,44].

The chronic stage can be summarized with the rule of “6 D’s” that represent joint distention, destruction, dislocation, disorganization, debris, and increased bone density [45].

Three key radiographic parameters are commonly used to assess the extent of deformation [44,46]: (1) Meary’s angle (MA): Also referred to as the talar-first metatarsal angle, this measurement represents the angle formed between the longitudinal axis of the talus and the first metatarsal. A normal Meary’s angle is approximately 0°, indicating proper alignment. (2) Calcaneal pitch angle (CP): This angle is defined as the inclination between the inferior border of the calcaneus and a line connecting the inferior weight-bearing point of the calcaneus to the base of the fifth metatarsal. The normal range for this angle is typically between 20° and 30°, reflecting the normal positioning of the calcaneus. (3) Cuboid height (CH): This measurement corresponds to the perpendicular distance from the plantar surface of the cuboid to a reference line extending from the calcaneal tuberosity’s plantar aspect to the plantar surface of the fifth metatarsal head. The average normal cuboid height is approximately 1.2 cm above this reference line.

## 4. Magnetic Resonance Imaging

Magnetic resonance imaging (MRI) is considered the gold standard for evaluation of soft tissues, ligaments, and bone marrow and their complications [47,48,49]. It is crucial for diagnosing CN, especially in the early stages when X-rays may not reveal any abnormalities. MRI also plays an important role in monitoring disease progression and identifying complications such as infection or osteomyelitis in more advanced stages [50,51,52].The images are typically obtained with a small field of view and thin sections (3–4 mm) to optimize spatial resolution. The imaging protocol should include T1 and short tau inversion recovery (STIR) or T2 fat-saturated sequences [45,53]. In axial and coronal projections, the ankle and tendons are assessed, while in sagittal projection, the metatarsophalangeal and interphalangeal joints, as well as the involvement of the midfoot, plantar surface, and calcaneus are evaluated [54].

The use of gadolinium-based contrast agents, while useful for determining the differences between active Charcot neuropathy and osteomyelitis, is debated due to its nephrotoxic effect. Patients with CNO are often diabetic patients with reduced renal function [47,55,56]. MR angiography (with or without contrast) can also be useful to assess distal vascularization, and MR neurography can be used to evaluate peripheral nerve damage [57].

**Figure 3:**
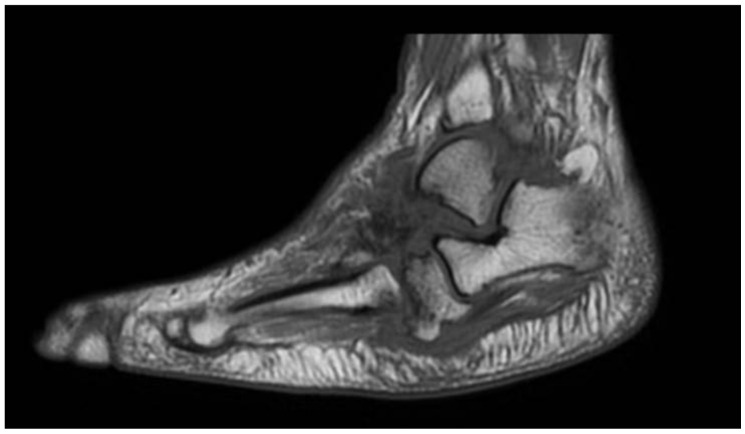
MRI (T1 FSE): Bone marrow edema is characterized by low signal intensity.

**Figure 4:**
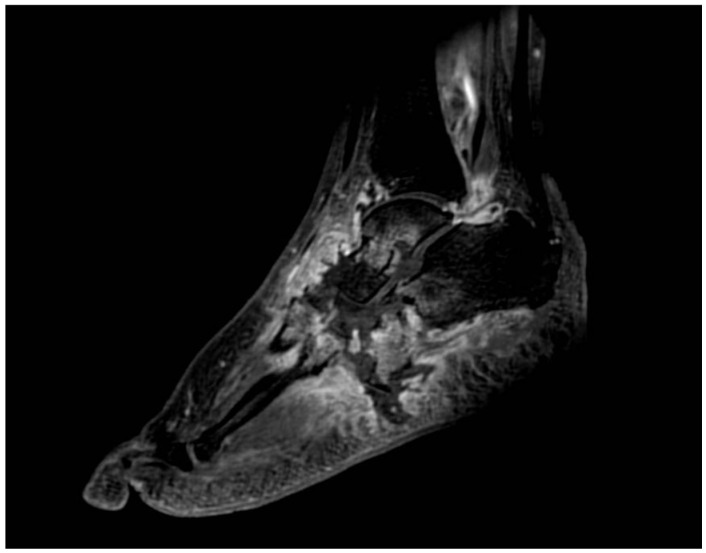
MRI (3D FSPGR + C): Subchondral bone marrow edema, joint effusions, and soft tissue inflammatory changes.

**Figure 5:**
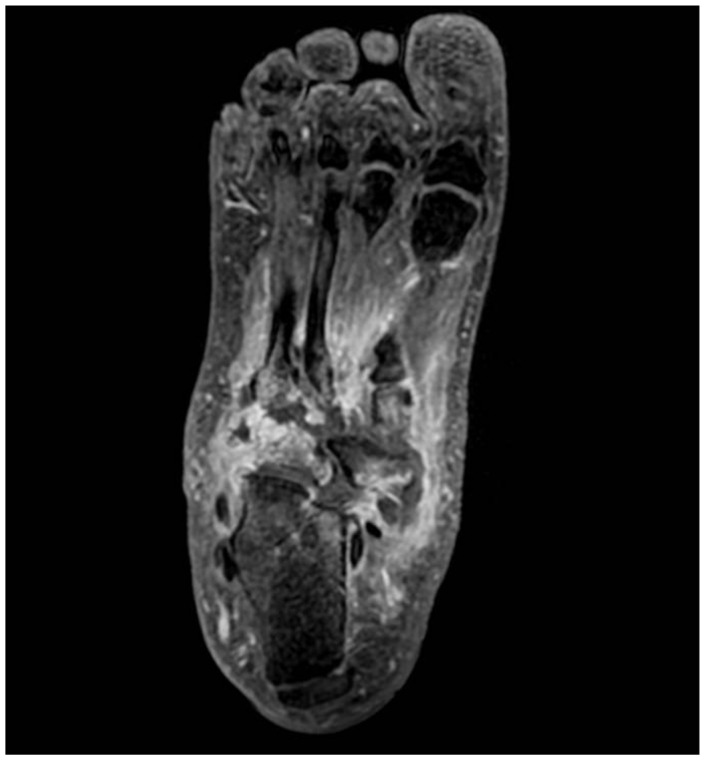
MRI (3D FSPGR + C): Subchondral bone marrow edema, joint effusions, and soft tissue inflammatory changes.

MRI represents the imaging method with the highest sensitivity in the early stages of CNO, which is fundamental for early diagnosis. Soft tissue edemas, joint effusions, fluid collections, and subchondral bone marrow edema with or without microfractures of involved joints are the most commonly seen findings in acute CNO [53,58]. Bone marrow edema is characterized by low signal intensity on T1-weighted images and high signal intensity on T2-weighted images (Figure 3, Figure 4 and Figure 5). Extension of edema throughout medullary bone is possible [12,59]. Bone-marrow enhancement is typically present predominantly in the subchondral region in gadolinium-enhanced studies [10,60].

In more advanced stages, edema, and enhancement are less evident on MRI [59]; rather, joint deformities with subluxations or bone dislocations are obvious [11]. In particular, there is superior and lateral subluxation of the metatarsals and rocker-bottom deformity, leading to the formation of calluses and ulcers at the level of the cuboid [45]. Subchondral cysts with defined margins (low signal on T1-weighted images and high signal on T2-weighted images), and intra-articular loose bodies are also present [12].

CNO in the active stage of the disease can clinically mimic (warm, reddened, swollen foot) a condition of osteomyelitis [61]. It is essential to distinguish between the two conditions because they require two extremely different treatments: immobilization/bracing/external fixation in one case, and antibiotic therapy/surgical debridement in the other [62].

Magnetic resonance imaging is decisive: it has a high sensitivity (77–100%) and high specificity (80–100%) for the diagnosis of osteomyelitis in the absence of neuropathic disease [63,64,65,66,67]. It also has a high negative predictive value of 98%. This means that when typical signs of osteomyelitis are absent, the condition can be effectively ruled out [47].

The analysis of radiological images reveals a focally decreased marrow signal intensity on T1, accompanied by an increased signal intensity on fat-suppressed T2 and STIR sequences, along with focal marrow enhancement on gadolinium-enhanced fat-suppressed T1-weighted images [68,69].

Skin ulceration and sinus tracts are often present, along with fluid collections that are larger than those seen in active CNO. The presence of cellulitis, soft tissue masses, and soft tissue abscesses is common, as well as cortical interruption. Additionally, there is a noted disappearance of subchondral cysts and intraarticular bodies [53,70,71,72,73].

Gadolinium contrast is instrumental in identifying fluid collections or abscesses, sinus tracts, and areas of devitalized tissue. The “ghost sign” observed in post-contrast images can indicate osteomyelitis coexisting with neuropathic arthropathy. This sign is characterized by bones appearing “dissolved” on T1-weighted images but regaining a more defined morphology on T2-weighted or contrast-enhanced imaging [60,74,75].

Even in MRI, acute CNO and osteomyelitis are similar, particularly due to the significant bone edema present; as a result, the differential diagnosis becomes more complicated [36,62] (Table 1). Both entities will exhibit subchondral bone marrow edema, joint effusions, and soft tissue inflammatory changes, characterized by high signal intensity on T2 fat-saturated or STIR images, and somewhat low signal intensity on T1 images [76]. In regions exhibiting discordant bone marrow signal alterations—characterized by elevated signal intensity on fluid-sensitive imaging sequences while maintaining a normal T1 signal—the intensity of the signal observed on fluid-sensitive sequences increases the likelihood of osteomyelitis being present or having the potential to develop in the future [50,62]. Additionally, there will be enhancement of bone and soft tissue following contrast administration [50].

The main difference, besides the absence of skin ulcers and the negativity of the host sign, is the localization of the bone edema [72]. In active CNO, it tends to be periarticular and affects multiple bones and joints, especially the tarsometatarsal and metatarsophalangeal joints. In osteomyelitis, it typically involves a single bone with greater involvement. The localization is variable, especially in the pressure surfaces of the foot, such as the metatarsal heads and the heel, in direct correlation with the skin ulcer and the sinus tract [47].

Patients with CNO are more susceptible to bacterial superinfection due to the metabolic and diabetic disorders they often experience, as well as an abnormal distribution of body weight on the plantar surface of the foot [77]. Therefore, it is essential to distinguish between acute neuropathy and infected neuropathy. In cases of infected neuropathy, the signs of infection will overlap with the signals of neuropathy [12].

## 5. Computed Tomography Scan

**Figure 6:**
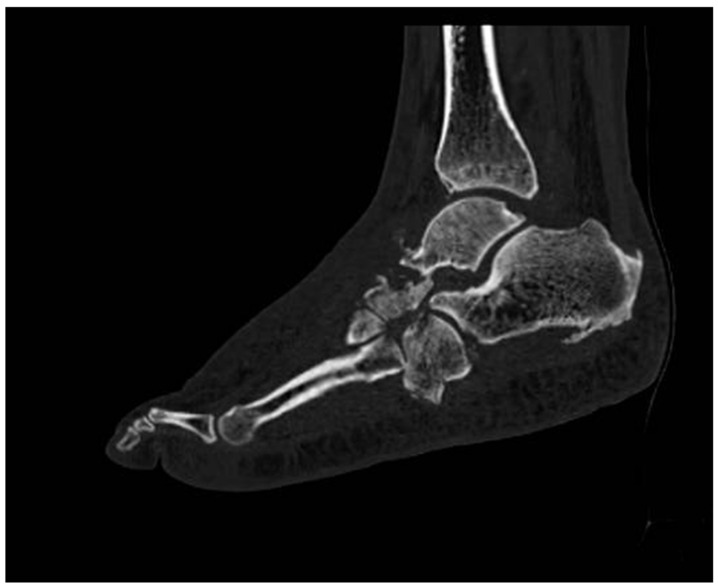
CT scan: Dislocations, bone fragmentation, and structural deformities.

**Figure 7:**
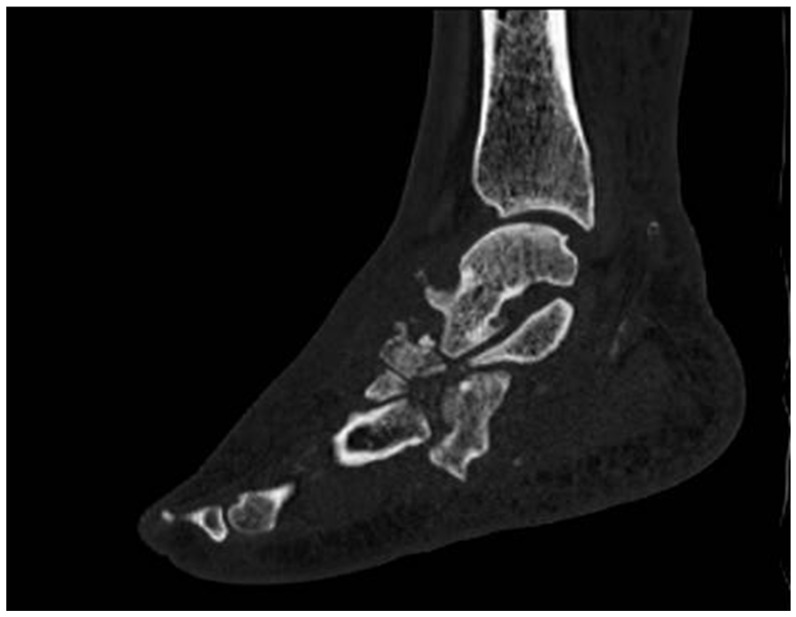
CT scan: Dislocations, bone fragmentation, and structural deformities.

Computed tomography scan (CT scan) imaging provides significant advantages in the evaluation and management of CNO, particularly in its advanced stages when inflammatory activity is diminished. These imaging modalities are instrumental in identifying and quantifying the anatomical changes induced by dislocations, bone fragmentation, and structural deformities (Figure 6 and Figure 7). Their utility extends to preoperative planning, guiding surgical interventions, and monitoring the outcomes of advanced treatments, such as the application of Ilizarov fixation systems, which require precise anatomical assessment to ensure successful outcomes [12,78].

Compared to MRI, CT offers superior visualization of cortical bone details. It is particularly effective in detecting cortical destruction, periosteal reactions, new bone formation, and small intramedullary gas foci—features that might be subtle or missed entirely on MRI scans. However, CT scans have limitations, as they are unable to reliably distinguish between different pathological processes such as purulence, granulation tissue, inflammation, or fibrosis. This lack of differentiation is a critical limitation when attempting to delineate active infection from sterile inflammation, areas where MRI holds an advantage due to its superior sensitivity for soft tissue and bone marrow changes [79,80,81].

Sagittal CT reconstructions are crucial for evaluating angular relationships between bones, aiding in the assessment of deformities, and facilitating detailed anatomical mapping. Additionally, transverse and coronal reconstructions are essential for visualizing the degree of dislocation in key joints, such as the tarsometatarsal or Lisfranc joint, which are commonly involved in CNO [29].

**Figure 8:**
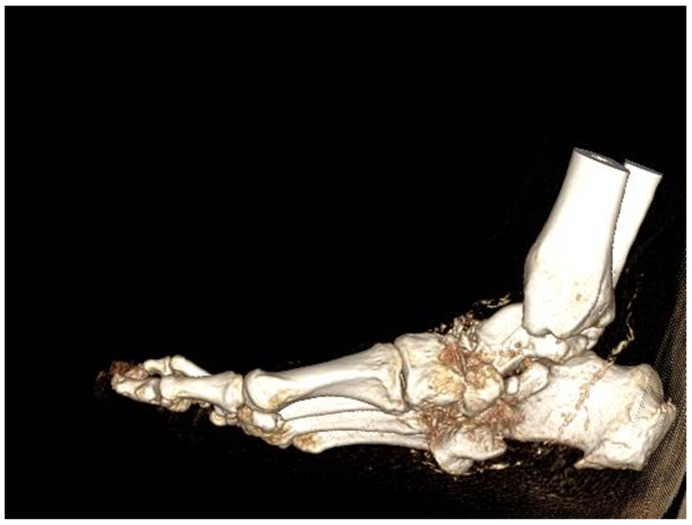
3D CT scan.

Three-dimensional reconstructions are indispensable in accurately assessing the extent of osseous involvement, particularly in cases where severe deformities, advanced structural deterioration, or extensive joint dislocations are present (Figure 8). These reconstructions provide critical insights into the spatial relationships between bone fragments, the degree of cortical destruction, and the overall anatomical integrity of the affected foot. Such detailed visualization plays a pivotal role in preoperative planning, allowing for precise corrective interventions, whether through conservative realignment strategies or complex surgical procedures, including arthrodesis and osteotomies [49].

Despite its significant advantages in characterizing bony architecture, CT remains a secondary diagnostic tool when differentiating diabetic neuroarthropathy from osteomyelitis. Although it can effectively complement clinical assessments and findings from other imaging modalities, it is not typically the first-line choice for distinguishing between these conditions. The primary limitation of CT in this context lies in its relatively lower sensitivity for detecting early inflammatory changes, such as bone marrow edema and soft tissue involvement, which are crucial markers for early CNO and infection.

MRI is generally preferred for the initial evaluation due to its superior ability to identify early pathological changes, including bone marrow abnormalities, soft tissue inflammation, and subtle subchondral fractures. MRI’s high sensitivity and specificity make it the gold standard for differentiating neuropathic bone changes from infectious processes, which is essential for guiding appropriate management strategies [50].

Nevertheless, CT retains a valuable role in cases where MRI findings are inconclusive or when MRI is contraindicated, such as in patients with certain metallic implants or severe claustrophobia. Additionally, its ability to provide high-resolution imaging of complex osseous structures makes it an important adjunctive tool in diagnostic completion, particularly in scenarios requiring precise anatomical delineation for surgical decision-making. As imaging technology continues to advance, the integration of multimodal approaches—combining CT, MRI, and nuclear imaging—may further enhance diagnostic accuracy, ensuring a more comprehensive assessment of diabetic foot complications. Ultimately, the judicious selection of imaging techniques based on clinical presentation and individual patient factors remains paramount in optimizing outcomes and preventing disease progression [54,82]. A study by Bouman et al. [83] that evaluated the diagnostic value of bone marrow edema (BME) detection on virtual noncalcular images (VNCa) from dual-energy CT (DECT) in people with diabetes and suspected Charcot Neuro-Osteoarthropathy (CNO) was very useful. The study found in 32 patients, 11 of whom had severe CNO, that midfoot CT values were significantly higher in the group with CNO than in the group without CNO. BME detection showed high sensitivity (100%) and moderate specificity (71.4%) for identifying active CNO. The results suggest that detection of BME on VNCa images could help diagnose active CNO in diabetic patients.

## 6. Nuclear Imaging

Lauri et al. have shown that nuclear medicine imaging is useful for distinguishing between CNO and osteomyelitis. While all imaging methods provide good sensitivity for conditions related to infection, they are quite expensive and expose patients to high doses of radiation [84]. For this reason, these imaging techniques are reserved for the most challenging cases and are typically considered a second-line option after MRI.

Bone scintigraphy (technetium-99m methylene diphosphonate (Tc-MDP) bone scan) can reveal signs such as focal hyperperfusion, focal hyperemia, and increased focal uptake of tracer material. These findings could be interpreted as indicators of osteomyelitis. However, similar results can also occur in other conditions, particularly in diabetic patients with foot problems. For instance, fractures, chronic soft tissue infections, and neuropathic joints can also show positive results on three-phase bone scans, which reduces the specificity of this diagnostic method [85].

A study on the use of SPECT/CT (Single Photon Emission Computed Tomography/Computed Tomography) in patients with swollen and hot feet but normal radiographs, often referred to as stage 0 showed showed distinct bone pathology in stage 0 CNO classified into three groups based on CT and SPECT findings: fractures with tracer uptake, bone abnormalities with tracer uptake, and normal CT but with focal tracer uptake. The CT component identified fractures in 59% of patients with early treatment. The study supports the use of SPECT/CT for early diagnosis of CNO and emphasizes the need for urgent treatment to prevent progression [86].

While a positive scan result does not definitively confirm the presence of osteomyelitis, a negative result offers a high level of certainty that an infection is not present in non-neuropathic joints, demonstrating the strong negative predictive value of bone scans [87]. Research has shown that using radiolabeled leukocytes in white blood cell (WBC) scans provides a highly sensitive method for diagnosing osteomyelitis, achieving a sensitivity rate of 97% [87]. However, in cases of Charcot Foot, false positives may arise even when there is no infection present. This can occur because leukocytes can accumulate at the site of microfractures, which are not visible on X-rays [88]. Fluorine-18-2-fluoro-2-deoxy-D-glucose (FDG-PET/CT) plays a potentially significant role in diagnosing deep soft-tissue infections and osteomyelitis, as well as differentiating neuropathic arthropathy [89,90]. Furthermore, FDG-PET/CT is suitable for evaluating patients with metal implants that might compromise the accuracy of MRI or CT [90]. Its high resolution provides an advantage over single-photon emitting tracers, particularly in accurately localizing radiotracer uptake in the bones of the distal forefoot, the most common site of diabetic foot infections [91]. The fused imaging capabilities of FDG-PET/CT enable accurate differentiation between osteomyelitis and soft-tissue infections [92,93]. A very useful study by Pickwell et al. [94] in this regard showed how F-18 FDG PET/CT reports increased F-18 FDG uptake in 25 areas of soft tissue and bone in nine patients, indicating inflammation, with no concomitant bone abnormalities on CT scan, suggesting an inflammatory origin with secondary bone resorption, potentially due to an altered neurogenic inflammatory response from small fiber neuropathy.

## 7. Conclusions

The radiological assessment of CNO in the diabetic foot plays a fundamental role in ensuring a timely diagnosis, accurate staging, and effective management of this complex and progressive condition. Given the severe consequences associated with delayed or inaccurate diagnosis—particularly the significant challenge of differentiating CNO from osteomyelitis—leveraging a combination of advanced imaging modalities is essential to optimize patient outcomes and reduce the risk of misdiagnosis.

Its wide availability and cost-effectiveness characterize plain radiography as an initial screening tool and often already useful for a diagnosis of CNO. Weight-bearing radiographs provide valuable insights into structural abnormalities, joint misalignment, and the progression of osseous changes over time. However, their diagnostic utility in the early, acute stages of CNO is significantly limited due to poor sensitivity in detecting subtle bone marrow alterations, early subchondral fractures, or soft tissue involvement. As a result, more sophisticated imaging techniques are required to facilitate early and accurate detection.

MRI and CT have revolutionized the diagnostic landscape by offering unparalleled visualization of both osseous and soft tissue changes. MRI, in particular, stands as the gold standard for early diagnosis due to its superior ability to detect bone marrow edema, soft tissue inflammation, and incipient subchondral fractures. Its high sensitivity and specificity allow for a clear distinction between neuropathic changes and infectious processes, a crucial factor in guiding appropriate clinical interventions. The use of contrast-enhanced MRI further enhances diagnostic precision by identifying areas of active inflammation and vascular compromise, making it indispensable in ambiguous cases.

Although less sensitive to acute inflammatory changes, CT imaging remains invaluable in characterizing advanced anatomical deformities, cortical destruction, and bone fragmentation. The capability of CT to provide detailed three-dimensional reconstructions significantly aids in surgical planning by offering precise visualization of bone fragments, joint instability, and spatial relationships within the affected foot. This level of anatomical clarity is particularly beneficial for preoperative assessment and in cases where severe structural compromise necessitates reconstructive procedures. Beyond conventional imaging, nuclear medicine techniques—including bone scintigraphy, radiolabeled leukocyte imaging, and FDG-PET/CT—offer an additional layer of diagnostic specificity, particularly in cases where conventional imaging yields inconclusive results. FDG-PET/CT, with its high spatial resolution and ability to detect metabolic activity associated with infection, is particularly valuable in distinguishing osteomyelitis from CNO, even in complex clinical scenarios such as the presence of metallic implants or postoperative changes. However, due to the higher costs, radiation exposure, and limited accessibility of these modalities, their use is typically reserved for second-line investigations in diagnostically challenging cases. Looking ahead, addressing current diagnostic limitations remains a critical priority in improving patient care. The standardization of imaging protocols and the development of integrated diagnostic algorithms that synthesize clinical, radiological, and laboratory findings will be essential for enhancing diagnostic accuracy and consistency across healthcare settings. Moreover, emerging imaging biomarkers and novel techniques, such as diffusion-weighted MRI and dynamic contrast-enhanced imaging, hold significant promise for refining early detection capabilities, monitoring disease progression, and assessing treatment efficacy. In conclusion, the comprehensive management of CNO in the diabetic foot requires a multidisciplinary approach that integrates advanced imaging with clinical expertise. As the field continues to evolve, the adoption of innovative imaging modalities, the implementation of standardized diagnostic frameworks, and ongoing advancements in radiological technology will be pivotal in improving diagnostic precision, minimizing complications, and ultimately enhancing the quality of care for affected patients.

## Figures and Tables

**Table 1:** Osteomyelitis and CNO comparison.

Characteristic	Osteomyelitis	Active CNO (Charcot Neuro-Osteoarthropathy)
Bone Edema	Typically in a single bone area	Periarticular, affecting multiple bones and joints
Localization	Often in pressure surfaces of the foot (metatarsal heads, heel), correlated with skin ulcers and sinus tracts	Periarticular, especially tarsometatarsal, metatarsophalangeal joints and tibio-tarsal joints
MRI Findings	Focally decreased marrow signal intensity on T1, increased signal on fat-suppressed T2 and STIR, focal marrow enhancement on gadolinium-enhanced T1-weighted images	Subchondral bone marrow edema, joint effusions, soft tissue inflammatory changes (high signal on T2, low on T1)
Signal Changes on Fluid-sensitive Imaging	Increased signal intensity on fluid-sensitive sequences with normal T1 signal	Increased signal intensity on fluid-sensitive sequences with normal T1 signal
Edema Signal Distribution	Localized to a single area with greater involvement	Periarticular, affecting multiple bones/joints
Contrast Enhancement	Enhancement in both bone and soft tissues after contrast administration	Enhancement in both bone and soft tissues after contrast administration
Presence of Skin Ulcers and Sinus Tracts	Skin ulcers and sinus tracts often present, along with large fluid collections	Absence of skin ulcers and sinus tracts, no significant fluid collections
Risk of Bacterial Superinfection	High, especially in patients with metabolic disorders or diabetes	More susceptible to bacterial superinfection in some cases, especially with neuropathy
Clinical Presentation	Signs of infection (fever, pain, redness) with abscesses and cellulitis	Signs of neuropathy with potential overlap in infected neuropathy
